# Supplementary material for: Role and mechanism of NCAPD3 in promoting malignant behaviors in gastric cancer
Source: Front Pharmacol. 2024 Apr 22;15:1341039. doi: 10.3389/fphar.2024.1341039 (PMC11070777; doi:10.3389/fphar.2024.1341039)
Supplement: Supplementary file 11 [file DataSheet2.ZIP › GSEA/GO_CC/my_analysis.Gsea.1599461408869/GO_CELL.html]

Details for gene set GO\_CELL[GSEA]

|  || Dataset | filtered\_dataset.sample\_info.cls#WT\_versus\_NCAPD3\_MUT |
| Phenotype | sample\_info.cls#WT\_versus\_NCAPD3\_MUT |
| Upregulated in class | NCAPD3\_MUT |
| GeneSet | GO\_CELL |
| Enrichment Score (ES) | -0.23110956 |
| Normalized Enrichment Score (NES) | -1.6221249 |
| Nominal p-value | 0.010666667 |
| FDR q-value | 0.11930183 |
| FWER p-Value | 0.709 |
Table: GSEA Results Summary

  

Fig 1: Enrichment plot: GO\_CELL      
 Profile of the Running ES Score & Positions of GeneSet Members on the Rank Ordered List

  

| SYMBOL | TITLE | RANK IN GENE LIST | RANK METRIC SCORE | RUNNING ES | CORE ENRICHMENT || 1 | 7052 | TGM2 | 17 | 1.045 | 0.0129 | No |
| 2 | 51134 | CEP83 | 19 | 1.038 | 0.0376 | No |
| 3 | 3954 | LETM1 | 47 | 0.924 | 0.0400 | No |
| 4 | 56288 | PARD3 | 99 | 0.790 | 0.0213 | No |
| 5 | 26272 | FBXO4 | 133 | 0.767 | 0.0155 | No |
| 6 | 79789 | CLMN | 160 | 0.717 | 0.0136 | No |
| 7 | 10161 | LPAR6 | 163 | 0.711 | 0.0295 | No |
| 8 | 528 | ATP6V1C1 | 254 | 0.625 | -0.0223 | No |
| 9 | 10464 | PIBF1 | 264 | 0.622 | -0.0138 | No |
| 10 | 23341 | DNAJC16 | 276 | 0.613 | -0.0069 | No |
| 11 | 538 | ATP7A | 305 | 0.588 | -0.0134 | No |
| 12 | 79443 | FYCO1 | 313 | 0.586 | -0.0043 | No |
| 13 | 7220 | TRPC1 | 328 | 0.576 | -0.0007 | No |
| 14 | 94107 | TMEM203 | 333 | 0.574 | 0.0104 | No |
| 15 | 1203 | CLN5 | 358 | 0.562 | 0.0063 | No |
| 16 | 1080 | CFTR | 366 | 0.557 | 0.0147 | No |
| 17 | 23315 | SLC9A8 | 398 | 0.537 | 0.0047 | No |
| 18 | 147007 | TMEM199 | 410 | 0.533 | 0.0095 | No |
| 19 | 805 | CALM2 | 423 | 0.518 | 0.0132 | No |
| 20 | 801 | CALM1 | 424 | 0.518 | 0.0259 | No |
| 21 | 5295 | PIK3R1 | 467 | 0.493 | 0.0067 | No |
| 22 | 4987 | OPRL1 | 477 | 0.487 | 0.0119 | No |
| 23 | 8454 | CUL1 | 492 | 0.477 | 0.0131 | No |
| 24 | 51112 | TRAPPC12 | 504 | 0.471 | 0.0164 | No |
| 25 | 3607 | FOXK2 | 522 | 0.461 | 0.0150 | No |
| 26 | 56652 | C10orf2 | 531 | 0.457 | 0.0202 | No |
| 27 | 90639 | COX19 | 535 | 0.454 | 0.0291 | No |
| 28 | 8195 | MKKS | 542 | 0.449 | 0.0356 | No |
| 29 | 2021 | ENDOG | 648 | 0.400 | -0.0329 | No |
| 30 | 23212 | RRS1 | 681 | 0.387 | -0.0472 | No |
| 31 | 5311 | PKD2 | 708 | 0.372 | -0.0575 | No |
| 32 | 404636 | FAM45A | 764 | 0.341 | -0.0902 | No |
| 33 | 4869 | NPM1 | 772 | 0.336 | -0.0872 | No |
| 34 | 3093 | UBE2K | 795 | 0.326 | -0.0956 | No |
| 35 | 57181 | SLC39A10 | 811 | 0.313 | -0.0991 | No |
| 36 | 1605 | DAG1 | 823 | 0.304 | -0.0999 | No |
| 37 | 7037 | TFRC | 824 | 0.303 | -0.0925 | No |
| 38 | 3146 | HMGB1 | 831 | 0.295 | -0.0897 | No |
| 39 | 6558 | SLC12A2 | 839 | 0.276 | -0.0882 | No |
| 40 | 4502 | MT2A | 863 | -0.277 | -0.0986 | No |
| 41 | 7296 | TXNRD1 | 877 | -0.289 | -0.1012 | No |
| 42 | 2150 | F2RL1 | 887 | -0.304 | -0.1005 | No |
| 43 | 9181 | ARHGEF2 | 902 | -0.326 | -0.1029 | No |
| 44 | 3949 | LDLR | 929 | -0.353 | -0.1137 | No |
| 45 | 4088 | SMAD3 | 930 | -0.353 | -0.1050 | No |
| 46 | 2317 | FLNB | 936 | -0.360 | -0.0999 | No |
| 47 | 5621 | PRNP | 970 | -0.387 | -0.1151 | No |
| 48 | 1657 | DMXL1 | 1080 | -0.462 | -0.1851 | No |
| 49 | 1604 | CD55 | 1090 | -0.468 | -0.1803 | No |
| 50 | 857 | CAV1 | 1098 | -0.475 | -0.1739 | No |
| 51 | 2729 | GCLC | 1123 | -0.497 | -0.1797 | No |
| 52 | 472 | ATM | 1193 | -0.568 | -0.2172 | Yes |
| 53 | 9709 | HERPUD1 | 1202 | -0.578 | -0.2090 | Yes |
| 54 | 27 | ABL2 | 1218 | -0.592 | -0.2057 | Yes |
| 55 | 26137 | ZBTB20 | 1244 | -0.622 | -0.2091 | Yes |
| 56 | 5106 | PCK2 | 1252 | -0.640 | -0.1987 | Yes |
| 57 | 63971 | KIF13A | 1256 | -0.645 | -0.1852 | Yes |
| 58 | 80201 | HKDC1 | 1289 | -0.681 | -0.1924 | Yes |
| 59 | 57835 | SLC4A5 | 1319 | -0.736 | -0.1960 | Yes |
| 60 | 6262 | RYR2 | 1323 | -0.741 | -0.1801 | Yes |
| 61 | 7779 | SLC30A1 | 1332 | -0.755 | -0.1676 | Yes |
| 62 | 56605 | ERO1B | 1340 | -0.778 | -0.1537 | Yes |
| 63 | 1649 | DDIT3 | 1341 | -0.779 | -0.1347 | Yes |
| 64 | 221937 | FOXK1 | 1344 | -0.785 | -0.1169 | Yes |
| 65 | 9990 | SLC12A6 | 1379 | -0.922 | -0.1197 | Yes |
| 66 | 5156 | PDGFRA | 1383 | -0.932 | -0.0992 | Yes |
| 67 | 256979 | SUN3 | 1386 | -0.954 | -0.0773 | Yes |
| 68 | 8867 | SYNJ1 | 1387 | -0.979 | -0.0533 | Yes |
| 69 | 493 | ATP2B4 | 1401 | -1.166 | -0.0345 | Yes |
| 70 | 760 | CA2 | 1410 | -1.654 | -0.0000 | Yes |
Table: GSEA details [plain text format]

  

Fig 2: GO\_CELL      
 Blue-Pink O' Gram in the Space of the Analyzed GeneSet

  

Fig 3: GO\_CELL: Random ES distribution      
 Gene set null distribution of ES for **GO\_CELL**
